# Effects of CO_2_ and liquid digestate concentrations on the growth performance and biomass composition of *Tetradesmus obliquus* and *Chlorella vulgaris* microalgal strains

**DOI:** 10.3389/fbioe.2024.1459756

**Published:** 2025-01-09

**Authors:** Ángela Sánchez-Quintero, Aurélien Parsy, Amandine Adrien, Lea Spitzer, Javier Jiménez-Lamana, Susana C. M. Fernandes, Jean-Baptiste Beigbeder

**Affiliations:** ^1^ APESA Pôle valorisation, Montardon, France; ^2^ Université de Pau et des Pays de l’Adour, E2S UPPA, IPREM, CNRS, Pau, France; ^3^ MANTA—Marine Materials Research Group, Université de Pau et des Pays de l’Adour, E2S UPPA, Anglet, France

**Keywords:** microalgae, liquid digestate, CO_2_, market regulation, biostimulants, biomass composition, metal content

## Abstract

This study evaluated the growth performance of *Tetradesmus obliquus* and *Chlorella vulgaris* microalgae cultivated in diluted liquid digestate supplemented with CO_2_, comparing their efficiency to that of a conventional synthetic media. The presence of an initial concentration of ammonium of 125 mg N-NH_4_^+^.L^-1^ combined with the continuous injection of 1% v/v CO_2_ enhanced the optimal growth responses and bioremediation potential for both strains in 200-mL cultures. In 6-L flat panel reactors, *T. obliquus* exhibited superior biomass production, achieving a final biomass concentration of 1.29 ± 0.06 g.L^-1^, while *C. vulgaris* reached only 0.36 ± 0.02 g.L^-1^. Both strains effectively contributed to the bioremediation of the digestate-based culture media, with up to 100% of N-NH_4_^+^, 50% of COD, and 55% of P-PO_4_^3-^ removals. The high nitrogen levels in the digestate-based medium significantly increased protein content, with 46.21% ± 3.98% dry weight (DW) for *T. obliquus* and 44.17% ± 2.24% DW for *C. vulgaris* as compared to the microalgae cultivated in commercial media. Additionally, the metal content of the microalgal biomass was analyzed to assess its potential use as biostimulants in compliance with European regulations. While chromium concentrations slightly exceeded regulatory thresholds in both strains, the levels of other metals remained within permissible limits.

## Graphical Abstract

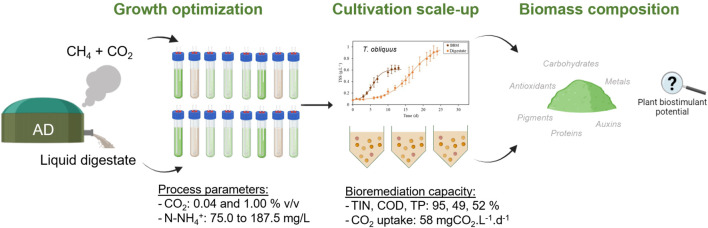

## 1 Introduction

Anaerobic digestion (AD) is a well-established biological process that effectively treats a wide range of organic wastes, while simultaneously producing sustainable energy (Chen W. et al., 2023). In the absence of oxygen, various microorganisms decompose organic matter into biogas, primarily composed of methane (CH_4_) and carbon dioxide (CO_2_) (Uddin and Wright, 2022). Given the European Union’s goal to reduce greenhouse gas (GHG) emissions by 2030 and achieve net-zero emissions by 2050, AD is becoming increasingly relevant (Bumharter et al., 2023). Currently, over 18000 biogas plants are operational across Europe (Bolzonella et al., 2023), with France positioned as the second country in terms of biomethane potential from AD by 2030 and projected to lead by 2050 (European Biogas Association, 2022).

In addition to biogas, digestate is the main co-product of the AD process, which can be separated into liquid and solid phases (Uddin and Wright, 2022). The liquid phase represents a turbid, nutrient-rich solution, mainly composed of nitrogen, phosphorus, and essential minerals (Sobolewska et al., 2022). Although the AD digestate is mainly used as a fertilizer in agriculture due to its beneficial agronomic and amending properties (European Biogas Association, 2023), concerns have emerged regarding its potential environmental impacts on soil health and microbial ecosystems (Karimi et al., 2022). Furthermore, the application of the AD digestate in agricultural practices is governed by stringent regulations, underscoring the urgent need for safer and more effective agricultural strategies (Wang et al., 2023). Even if several strategies are already available for the proper management of digestate, including struvite precipitation, membrane separation, or ammonium stripping, there is an urgent need to explore innovative and environmentally friendly alternatives (Wang et al., 2023).

As the AD sector continues to develop, researchers investigate new strategies for managing digestate and biogenic CO_2_ (Chozhavendhan et al., 2023). Microalgae present promising solutions for both challenges. They efficiently assimilate a variety of nutrients found in the liquid digestate (Leca et al., 2023) and can utilize CO_2_ as an inorganic carbon source through their autotrophic or mixotrophic metabolism (Yang et al., 2022). Beyond their bioremediation potential, microalgal biomass can be used for the production of bioproducts and biofuels, with applications spanning several sectors, including cosmetics, energy, and particularly, agriculture, where they can function as biostimulants and biofertilizers (Barbosa et al., 2023).

Cultivating microalgae in the digestate presents several challenges that must be addressed, including (i) the high turbidity of the digestate; (ii) ammonia/ammonium toxicity; and (iii) the presence of organic and biological contaminants (Chong et al., 2022). Various microalgal strains have been successfully cultivated in the digestate while managing these factors. Notable examples include *Tetradesmus obliquus,* which was cultivated by diluting the AD liquid digestate from zootechnical wastes and vegetable waste (Massa et al., 2017); *Chlorella vulgaris,* which thrived in pig manure AD digestate pre-treated by indigenous bacteria (Gu et al., 2021); and *Arthrospira platensis,* which was grown by diluting AD liquid digestate with geothermal water (Leca et al., 2024).

For producing biostimulants in sustainable agriculture, these microalgal strains have also been cultivated in various wastewater sources beyond AD liquid digestate. Examples include piggery wastewater (Ferreira et al., 2021), municipal wastewater (Carneiro et al., 2021), poultry wastewater (Viegas et al., 2021), and cheese whey wastewater (Zapata et al., 2021). It is important to note that the biochemical composition of microalgae is influenced by the characteristics of the culture media, as well as the culture conditions (photoperiod, strains, temperature, pH, etc.) (Menezes et al., 2016).

In this study, the growth capacity, nutrient uptake efficiency, and biomass composition of two different microalgal strains, *T. obliquus* and *C. vulgaris*, were compared, using different compositions of AD liquid digestate and concentrations of CO_2_. Bold Basal Medium (BBM) served as a conventional culture medium for benchmarking the growth performance of both strains against the digestate-based media. Initially, we conducted a screening in 200-mL cultures using various dilutions of the AD digestate mixed with tap water, along with an injection of air with 0.04% or 1% v/v CO_2_. The best-performing conditions were then scaled up to a 6-L flat panel reactor. Finally, the harvested biomass was fully characterized to determine its potential application as a plant biostimulant.

## 2 Materials and methods

### 2.1 Sampling of the anaerobic digestate

The AD digestate was sampled from the industrial biogas plant “Asson Bioenergie” located in Asson, France, in 2022. This facility injects approximately 112 Nm^3^.h^-1^ of biomethane into the natural gas network. The AD plant primarily utilizes local agricultural residues, including energy crops and manure, as principal feedstock, processing approximately 30 - 37 kt·y^1^ of organic matter.

The raw digestate was collected directly from the anaerobic digester and filtered through a series of sieves with decreasing cut-off points. The filtration process began with a 2-mm sieve, followed by 1-mm, 300-µm, and finally, 50-µm sieves, to recover the liquid filtrate. The resulting AD liquid digestate was then stored at −20°C for further analysis. The raw and liquid AD digestates were characterized following the methodology outlined in Section 2.2 (Supplementary Tables S1, S2, respectively).

### 2.2 Digestate characterization

The concentrations of N-NH_4_^+^ (APHA 4500-NH_3_ F), COD (APHA 522 D), N-NO_3_^-^ (ISO 8466-1), N-NO_2_^-^ (ISO11905-1), and P-PO_4_^3-^ (APHA 4500-PC) in the liquid digestate were determined using conventional reagent kits (Spectroquant^®^) and a photoLab S6 photometer (WTW, Xylem Analytics, Germany), following established standard protocols (APHA, 1999. *Standard methods for the examination of water and wastewater*, American Public Health Association). Total volatile fatty acid (VFA) content was assessed by gas chromatography (GC-FID 7890B, Agilent Technologies, Germany). The pH was measured using a pH meter (WTW, France), while conductivity was determined using a conductivity meter (WTW, France). Turbidity was measured using a turbidimeter according to ISO 7027 standards (Hanna instruments, France). The mineral and metal composition of the liquid digestate were evaluated by an external laboratory (Wessling, France) according to ISO 17294-2 standards.

For the raw digestate, the total Kjeldahl nitrogen (TKN), phosphorus (P-P_2_O_5_), potassium (K^+^) sodium (Na^+^), calcium (Ca^2+^), magnesium (Mg^2+^), and sulfur (S-SO_4_^2−^) concentrations were determined in duplicate by another external laboratory (Aurea, France) according to the NF EN ISO 11885 standard (Supplementary Table S1).

### 2.3 Microalgal inoculum preparation

*T*. *obliquus* BEA 0140B and *C*. *vulgaris* AC150 strains were provided by the Banco Español de Algas and Algobank CAEN, respectively. Prior to experimentation, both strains were cultured in 5-L glass bottles until they reached the stationary phase. The cultures were maintained in a synthetic commercial medium, BBM, purchased from Sigma-Aldrich, composed of the following concentrations (mg.L^-1^): 11.42 H_3_BO_3_, 25.0 CaCl_2_.2H_2_O, 0.49 Co(NO_3_)_2_.6 H_2_O, 1.57 CuSO_4_.5H_2_O, 50.0 EDTA, 4.98 FeSO_4_.7H_2_O, 75.0 MgSO₄.7 H₂O, 1.44 MnCl_2_.4H_2_O, 0.71 MoO_3_, 0.003 NiCl_2_.6H_2_O, 31.0 KOH, 0.003 KI, 175.0 KH_2_PO_4_, 75.0 K_2_HPO_4_, 25.0 NaCl, 250.0 NaNO_3_, 0.002 Na_2_SeO_3_, 0.001 SnCl_4_, 0.0022 V_2_(SO_4_)_3_.3H_2_O, and 8.82 ZnSO₄.7H₂O. The microalgal inocula were mixed by continuous injection of air and subjected to 16 h of light and 8 h of darkness at room temperature.

### 2.4 Microalgal culture experiments

#### 2.4.1 Optimal dilution screening

For the screening, both microalgal strains were cultivated individually in 250-mL tubes, maintaining a constant usable volume of 200 mL to determine the optimal growth and treatment conditions for subsequent scaling up to 6 L. Various dilutions of the AD liquid digestate in tap water were employed as the culture medium, specifically at a Df of 10, 15, 20, and 25 (Table 1). Commercial BBM was tested to confirm the quality of the inoculum. Each culture condition was subjected to an injection of 1% v/v CO_2_ at a flow rate of 0.4 NL.min^-1^, which was compared against an injection of air containing 0.04% v/v CO_2_ at the same flow rate.

**TABLE 1:** Culture medium conditions in the 200-mL tubes: dilution factors (Df) and concentrations of N-NH_4_^+^, P-PO_4_^3-^, and COD.

AD liquid digestate Df	N-NH_4_^+^ (mg.L^-1^)	P-PO_4_^3-^ (mg.L^-1^)	COD (mg.L^-1^)
10	187.50	9.90	1970
15	125.00	6.60	1313
20	93.75	4.95	985
25	75.00	3.96	788

The light intensity was maintained at 60 ± 5 µmol_hv._m^-2^.s^-1^ following a photoperiod of 16 h of light and 8 h of darkness. All the cultivations were performed in three independent biological replicates.

#### 2.4.2 Scaling up to 6-L flat panels

Based on the results obtained during the screening, a Df of 15 was selected for the assays conducted in 6-L bioreactors. Each bioreactor was supplied with a continuous injection of 1% v/v CO_2_ at a flow rate of 0.4 NL.min^-1^. For comparative analysis, both strains were also cultivated using BBM with the same CO_2_ injection rate. The light intensity was maintained at 146 ± 27 µmol_hv_.m^-2^.s^-1^ following a photoperiod of 16 h of light and 8 h of darkness. The final biomass was harvested in batches *via* centrifugation at 3500 rpm for 10 min using a ROTANTA 460 (Hettich Lab, France). The supernatant was discarded, and the solid biomass was washed twice with demineralized water before being collected and frozen at −20°C. All the experiments were performed in triplicate, and the results are presented as mean and standard deviation. For characterization, the microalgal biomass was dried using an Alpha 1-2 LDplus freeze-dryer (Christ, Germany). Subsequently, the dried biomass was ground using a mortar and pestle prior to analysis.

The culture media used in the 6-L experiments were characterized at both the beginning and end of the cultivation period following filtration through a 0.7-µm membrane. COD, TIN, N-NO_3_^-^, N-NO_2_^-^, N-NH_4_^+^, and P-PO_4_^3-^ concentrations were analyzed using the methods previously described.

#### 2.4.3 Growth monitoring

The growth of each culture was monitored by establishing growth curves through the measurement of the optical density (OD) at 680 nm every 24 h using a plate spectrophotometer (EPOCH, BioTek Instruments, France). For digestate-based media, the self-absorbance of the digestate was subtracted from the measurement of OD in order to avoid an overestimation of the algae production.

The pH of the cultures was measured each day during the algal cultivation experiment. The biomass concentration (*X*) (g.L^-1^) for *T. obliquus* (1) and *C. vulgaris* (2) was determined using the correlations in Equations 1, 2. To determine the total suspended solid (TSS) concentration, a specific volume of the microalgal suspension was filtered on a pre-weighed 0.7-µm dry filter (Whatman 47-mm GF/F glass microfiber filter) using a vacuum pump. After collecting the microalgal cells, the filters were then dried at 105°C for 24 h. The TSS was finally calculated by weight difference in relation to the volume of the filtered sample.

$$X\;\left(T.\;obliquus\right)=0.4645\times {OD}_{680}\;R^{2}=0.84,$$ (1)

$$X\;\left(C.\;vulgaris\right)=0.247\times {OD}_{680}+0.0149\;R^{2}=0.99,$$ (2)

where *X* represents the microalgal concentration in g.L^-1^ and R^2^ is the coefficient of determination.

#### 2.4.4 Biomass characterization

To establish the biomass composition of *T. obliquus* and *C. vulgaris* grown in both AD liquid digestate and synthetic BBM , carbohydrates, lipids, proteins (total and soluble), chlorophylls, and total phenols were quantified, among other parameters. All analyses were performed in triplicate, except for those on monomeric sugars, which were measured in duplicate. The majority of the results are expressed as % of elements in the dried biomass.

For carbohydrate quantification, a two-stage hydrolysis was conducted following the NREL protocol (Van Wychen and Laurens, 2016). In brief, 50 mg of freeze-dried biomass was added to 500 µL of H_2_SO_4_ 72% for 1 h under agitation at 350 rpm. Afterward, 14 mL of distilled water was added, and the mixture was heated to 120°C for 60 min. The resulting solution was then filtered through a 0.2-µm filter in preparation for high-performance liquid chromatography (HPLC) analysis, which was performed using the Agilent 1260 Infinity II model equipped with a Hi-Plex H column and a PL-HiPlex H guard column.

The crude lipids were analyzed using the modified gravimetric method of Bligh & Dyer (1959) at an external laboratory (GreenCoLab, Portugal). In this procedure, a 6:3 methanol/chloroform extraction was performed on the dried biomass. The resulting extracts were centrifuged, and the chloroform fraction was concentrated and dried overnight at 60°C.

The total nitrogen, hydrogen, and carbon content were measured by an external laboratory (GreenCoLab, Portugal) using a CHN elemental analyzer (Elementar Vario EL III, Germany) using 2 or 3 mg of biomass previously pounded in a ball mill (RETSCH MM 300). The protein content was determined according to the Dumas method, multiplying the nitrogen content by a specific conversion factor for microalgae of 6.25 (Templeton and Laurens, 2015).

Chlorophyll extraction was carried out using the Precellys Evolution Ozyme bead-beater (VK05 Bertin Corp tubes) with microtubes filled with 1 mL of methanol and 1 mg of dried biomass. The OD of supernatants was measured at 652 and 665 nm after centrifugation, and the chlorophyll content was calculated according to the *Handbook of Food Analytical Chemistry*, *Wiley Online Books*, 2004.

The mineral matter was measured by calcining and weighing the dried biomass at 550°C. Prior to this, the biomass was placed in an oven at 105°C for 24 h to measure the moisture content.

For the quantification of extractable proteins and phenolic compounds, 50 mg of freeze-dried microalgal biomass was homogenized using a Precellys Evolution Ozyme bead-beater in 2-mL tubes containing 0.5-mm glass beads (VK05 Bertin Corp.). The freeze-dried biomass was suspended in 1 mL of 1M NaOH (Huang et al., 2019; Naczk and Shahidi, 2004) and subjected to three cycles of 60 s at 65000 rpm with a 120-s pause between each cycle. Following homogenization, the contents of the tubes were centrifuged to separate the glass beads and remaining cell biomass from the supernatant, which contained the extracts for further analysis.

The extractable proteins were quantified using the Thermo Scientific™ BCA Protein Assay kit following the protocol outlined by Smith et al. (1985). Bovine serum albumin (BSA) was used as a standard, and the absorbance was measured at 562 nm using a microplate reader (Epoch, BioTek instruments).

For the quantification of phenolic compounds within the microalgal cells, a colorimetric method utilizing the Folin–Ciocalteu reagent was implemented to oxidize phenolic compounds, as described by Slinkard and Singleton (1977), with gallic acid serving as the standard. In this procedure, 100 µL of the supernatant was mixed with 500 µL of 2 N Folin–Ciocalteu reagent and 400 µL of Na_2_CO_3_ at 75 g.L^-1^. The mixtures were incubated for 20 min at 40°C with constant agitation at 450 rpm. The optical densities of the resulting solutions were then measured at 735 nm using a microplate reader (Epoch, BioTek instruments).

The Salkowski method (Gang et al., 2019) was used for the quantification of indole acetic acid (IAA) auxin. Samples were pounded with liquid nitrogen, and 50 mg of biomass was then incubated in 450 µL of methanol at −20°C overnight. After incubation, the extracts were centrifuged using a MiniSpin centrifuge (Eppendorf, France), and the resulting pellet was discarded. The Salkowski reagent was subsequently added to each sample at a 1:1 (v/v) ratio, followed by vortexing and incubation for 30 min at 30°C. Finally, the OD of the solutions was measured at 536 nm. Commercial IAA (Thermo Fisher Scientific) was used as the standard for calibration.

To determine the antioxidant capacity of both microalgal strains, ferric reducing antioxidant power (FRAP) assay was performed. The biomass was first ground with liquid nitrogen using a mortar and pestle. Microalgal suspensions (5 g.L^-1^) were extracted in ethanol overnight with agitation at 250 rpm, followed by centrifugation to remove the pellet and assess the antioxidant activity in the supernatant. Trolox, dissolved in ethanol, served as the standard.

For the FRAP assay, 200 µL of a working solution composed of 300 mM acetate buffer, 10 mM 2,4,6-tripyridyl-s-triazine in 40 mM HCl, and 20 mM ferric chloride hexahydrate (in a 10:1:1 ratio) was mixed with 60 µL of each extract in a 96-well plate. The mixture was incubated for 15 min at 37°C, and the optical density was measured at 593 nm using a Multiskan Sky Microplate Spectrophotometer (Thermo Fisher Scientific, Illkirch-Graffenstaden, France). The results of this assay are expressed as µmol of Trolox equivalents.mg of microalgal DW^-1^.

For the metal analysis, biomass samples of *T. obliquus* and *C. vulgaris* were subjected to a two-step digestion process prior to inductively coupled plasma mass spectrometry (ICP-MS) analysis. In the first step, 0.150 g of freeze-dried and ground biomass was pre-digested with 3 mL of HNO_3_ for 30 min, followed by the addition of 4 mL of H_2_O_2_ and further digestion for 1 h. The second step involved heating the samples at 85°C overnight, after which they were diluted with Milli-Q water at dilutions of 50, 100, or 1000, depending on the concentration of elements in the samples. An Agilent 7900 ICP mass spectrometer (Agilent, Tokyo, Japan) equipped with a collision cell (hydrogen/helium) was utilized for the analysis. Sample introduction was done using a concentric nebulizer and a Scott spray chamber. The H_2_ gas was injected at a flow rate of 5 mL min^−1^ to mitigate known interferences during isotope detection. The isotopes monitored included ^23^Na, ^24^Mg, ^31^P, ^39^K, ^42^Ca, ^52^Cr, ^54^Fe, ^55^Mn, ^60^Ni, ^63^Cu, ^64^Zn, ^75^As, ^112^Cd, ^201^Hg, and ^208^Pb. Multi-elemental calibration curves (0.1 ng mL^−1^ to 20 ng mL^−1^) were generated, yielding a coefficient of determination (R^2^) greater than 0.99.

### 2.5 Data processing

#### 2.5.1 Microalgal growth

The microalgal productivity of each culture was calculated using Equation 3, which determines the difference in the biomass concentration between the beginning and end of the culture period:

$$Pv=\frac{\left(X_{f}-X_{i}\right)}{t},$$ (3)

where

*Pv* is the global volumetric productivity (mg.L^-1^.d^-1^).

*X*_*f*_ and *X*_*i*_ are the microalgal concentrations (mg.L^-1^) at the end and beginning of the culture, respectively, and *t* is the culture time (d).

Growth parameters, including the maximum growth rate (d^-1^), lag phase time (d), and plateau reached during the stationary phase, were determined using the logistic model proposed by Zwietering et al. (1990), expressed in Equation 4:

$$\ln\;\left(\frac{X\left(t\right)}{X_{0}}\right)=\frac{A}{\left\{1+\exp\left[\frac{4\mu }{A}\left(\lambda -t\right)+2\right]\right\}},$$ (4)

where*t* is time (d),*X(t)* and *X*_*0*_ are the TSS concentrations at time t and day 0 (g.L^-1^), *A* is the plateau concentration reached in the stationary phase (unitless), *µ* is the maximum specific growth rate (d^-1^), and λ is the duration of the lag phase (d).

#### 2.5.2 Nutrient and CO_2_ uptake

The CO_2_ fixation rate was calculated according to Equation 5:

$$F_{{CO}_{2}}=C_{carbon}\cdot \;P_{v}\left(\frac{M_{{CO}_{2}}\;}{M_{C}}\right),$$ (5)

where*F*_*CO2*_ is the fixation rate of CO_2_ (mgCO_2_.L^-1^.d^-1^),*C*_*carbon*_ is the carbon content in the algal biomass (in wt%), which was theoretically set to 50% for the tube experiment and measured for the scale-up experiment,*M*_*CO2*_ is the molecular weight of CO_2_ (g.mol^-1^), *M*_*C*_ is the molecular weight of carbon (g.mol^-1^), and *P*_*v*_ is the volumetric productivity of the microalgae (mg.L^-1^.d^-1^).

A mass balance on the inorganic nitrogen was performed by comparing the nitrogen content of the final algal biomass and the removal of inorganic species in the digestate-based media. Consequently, the percentage of nitrogen assimilated by the microalgae (N_A_) during the cultivation was calculated according to Equation 6:

$$N_{A}=\frac{Pv\times C_{nitrogen}\times t}{\left({CN}_{0}-{CN}_{f}\right)\;}\times 100,$$ (6)

*C*_*nitrogen*_ is the nitrogen content in the algal biomass harvested during the cultivation assays in 6-L bioreactors (wt%), *t* is the duration of the culture, *P*_*v*_ is the volumetric productivity of the microalgae (mg.L^-1^.d^-1^), and *CN*_*0*_ and *CN*_*f*_ are the initial and final concentration of total inorganic nitrogen (TIN) species (sum of NH_4_^+^, NO_2_^−^, and NO_3_^−^) measured in the media, respectively.

The removal efficiency (%) and removal rate (mg.L^-1^.d^-1^) of different parameters including TIN, COD, and P-PO_4_^3-^ were calculated using Equations 7 and 8, respectively:

$$Removal\;efficiency=\frac{C_{0}-C_{f}}{C_{0}}\times 100,$$ (7)

$$Removal\;Rate=\frac{\left(C_{f}-C_{0}\right)}{t},$$ (8)

where*C*_*0*_ and *C*_*f*_ are the initial and final concentrations (mg.L^-1^) of the different parameters, respectively, and *t* is the duration of the culture.

#### 2.5.3 Statistical analyses

Statistical analyses were conducted using RStudio with the packages “ggplot2,” “ggthemes,” “multcompView,” and “dplyr.” To assess differences among the various parameters analyzed, one-way analyses of variance (ANOVA) were performed, followed by Tukey’s *post hoc* tests. A 95% confidence level (significance level of 0.05) was used, with *p*-values <0.05 considered statistically significant.

## 3 Results and discussion

### 3.1 Assessment of the optimal growth conditions in 200 mL cultures

**FIGURE 1:**
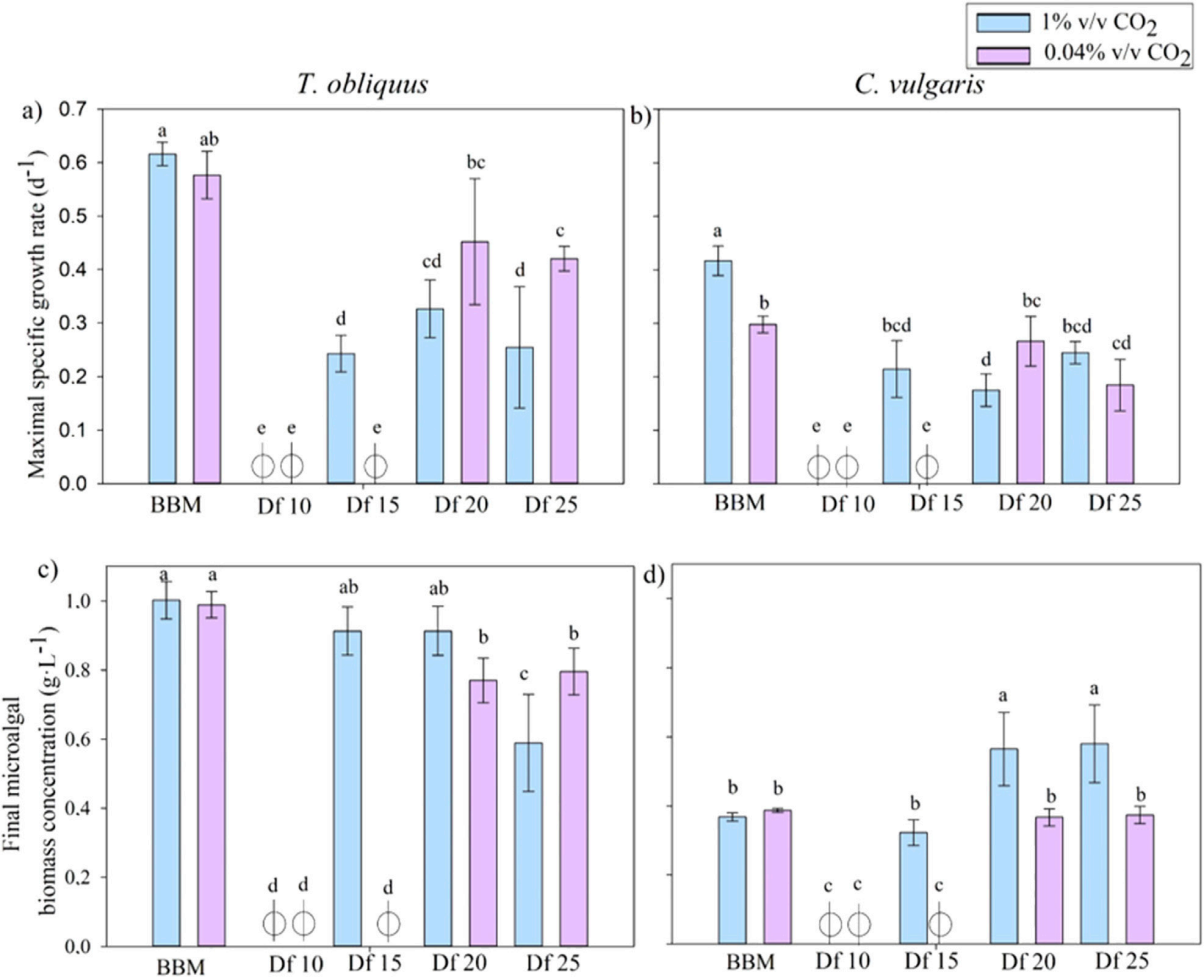
Comparison of the maximal specific growth rate (d ^-1^) of *T. obliquus* **(A)** and C. *vulgaris* **(B)** in different digestate dilutions (Df) with the injection of 1% v/v CO_2_ or with 0.04% v/v CO_2_ injection and microalgal concentrations (g.L^-1^) of *T. obliquus* **(C)** and C. *vulgaris* **(D)** at the end of the exponential phase. Statistical analyses were carried out for each microalgal strain separately. The same letter above bars indicates no significant difference between the tests (*p*-value >0.05).

A first culture screening was performed to determine the operational conditions (type of strain, quantity of digestate, and CO_2_ concentration) required to maximize the uptake of residual nutrients and CO_2_ and, at the same time, increase the biomass productivity. To do so, the digestates diluted at four different dilution factors (Df: 10, 15, 20, and 25) using 1% v/v CO_2_ or 0.04% v/v CO_2_ injection for each digestate Df were compared. A culture was also carried out in commercial BBM under the same conditions. Different growth parameters were assessed for each culture to determine the most appropriate culture condition, including (i) maximal growth rate and (ii) final biomass concentration (Figure 1).

Interestingly, no significant differences were observed in the maximum growth rates (a) or final biomass concentrations (c) of *T. obliquus* in the synthetic medium when comparing 1% and 0.04% v/v CO_2_ injections (Figure 1). The highest biomass concentration, reaching 1 ± 0.05 g.L^-1^, was recorded in the synthetic medium. In contrast, *C. vulgaris* demonstrated a notable increase of approximately 25% in the maximum growth rate (b) with the application of 1% compared to 0.04% v/v CO_2_ injection alone. However, there was no significant difference in the algal biomass concentration (d) between the two injection methods. Unlike *T. obliquus*, the highest final biomass concentration for *C. vulgaris* was achieved in the diluted digestate, where it reached approximately 0.4 g.L^-1^, rather than in the synthetic medium.

For *T. obliquus* (a), no growth was observed with a 10-fold dilution of the digestate, regardless of whether 1% or 0.04% v/v CO_2_ was injected in the cultures. Additionally, growth was absent with a 15-fold dilution under 0.04% v/v CO_2_. However, when the digestate was diluted 15-fold and supplemented with 1% v/v CO_2_, a growth rate of 0.24 ± 0.03 d^-1^ was achieved, representing 35% of the maximum specific growth rate recorded in the synthetic medium. This result was statistically comparable to those of the higher dilutions (20- and 25-fold) under CO_2_ injection. In contrast, growth under 0.04% v/v CO_2_ injection was only observed in the 20-fold and 25-fold diluted digestate, indicating that less liquid effluent and CO_2_ volumes were treated.

For *C. vulgaris* (b), growth was also not possible with a 10-fold dilution of the digestate. Similar to *T. obliquus*, growth was noted with the 15-fold dilution and 1% v/v CO_2_ injection, but not with 0.04% v/v CO_2_. The growth rate at the 15-fold dilution was not significantly different from that of other conditions, whether CO_2_ was injected or not, suggesting that less liquid effluent was treated in those instances. A maximum growth rate of 0.21 ± 0.05 d^-1^ was achieved using the 15-fold diluted digestate, which corresponds to 50% of the maximum growth rate observed in the synthetic medium.

These results are illustrated in Figures 1C, D, which depict the concentration of microalgae reached under each condition at the end of the exponential phase and the beginning of the stationary phase. A clear distinction can be observed between the injection of 0.04% and 1% v/v CO_2_. For *T. obliquus,* no significant difference was found between the 15- and 20-fold diluted digestate cultures. Conversely, for *C. vulgaris*, a significantly lower microalgal concentration was recorded in the 15-fold diluted digestate than in the concentrations achieved with the 20-fold and 25-fold dilutions. When compared to the synthetic medium, *T. obliquus* reached 91% of the final microalgal biomass (0.91 ± 0.07 g.L^-1^), while *C. vulgaris* achieved 88% (0.32 ± 0.04 g.L^-1^) under 1% v/v CO_2_ injection.

According to the literature, both *T. obliquus* and *C. vulgaris* exhibit enhanced growth efficiency with CO_2_ injection. Chaudhary et al. (2018) reported an approximately 8-fold increase in growth for both strains when 5% v/v CO_2_ was injected compared to air injection, which is likely due to increased carbon availability for microalgal assimilation. It is also important to consider the pH as CO_2_ injection tends to acidify the medium more than air alone, which may have influenced the proliferation of microalgae under these culture conditions (Elisabeth et al., 2021).

During the cultivation of *T. obliquus,* the pH decreased from 8.6 to 7.6 with the injection of 1% v/v CO_2_. In contrast, with 0.04% v/v CO_2_ injection, the pH fluctuated between 8.8 and 9.1. For cultures using the synthetic medium without the digestate, the pH remained stable between 6.9 and 7.2 under 1% v/v CO_2_ injection, while it ranged from 6.9 to 8.3 under 0.04% v/v CO_2_ injection. In the case of *C. vulgaris*, the pH under 1% v/v CO_2_ injection decreased from 8.1 to 7.6 throughout the culture period, whereas the cultures under 0.04% v/v CO_2_ injection exhibited pH values from 8.4 to 9.0. For synthetic medium cultures without the digestate, the pH remained between 7.0 and 7.2 with CO₂ injection and ranged from 7.4 to 8.6 with 0.04% v/v CO_2_ injection. Notably, in cultures containing the digestate, the pH variation was reduced as the amount of the digestate increased, indicating a buffering effect (Uggetti et al., 2014).

Table 2 shows that the rate of CO_2_ assimilation by microalgae was slightly higher with 1% v/v CO_2_ injection than with 0.04% v/v CO_2_ injection. While the CO₂ injection generally promotes microalgal growth, it remains uncertain whether the microalgae fully utilize all the injected CO₂ under these specific cultivation conditions. The most pronounced differences in growth between 0.04% and 1% v/v CO_2_ injections were observed under conditions with higher volumes of the digestate, which corresponded to elevated ammonium concentrations. Specifically, the 15-fold diluted digestate contained an initial concentration of 148 and 128 mg of N-NH_4_^+^.L^-1^ (Table 4). Despite the increased biomass production, the CO_2_ fixation rate did not exceed that observed with less diluted digestate or in the synthetic medium, where N-NH_4_^+^ concentrations were lower. This suggests that not all injected CO_2_ was assimilated by the microalgae for growth. The continuous aeration of the cultivation media probably led to the stripping of CO_2_ during the assays. Indeed, the low solubility of CO_2_ in water combined with the high aeration flow rate could have limited the CO_2_ transfer into the liquid phase.

**TABLE 2:** CO_2_ assimilation rates (mgCO_2_.L^-1^.d^-1^) for various dilution factors (Df) of liquid digestate using *T. obliquus* and *C. vulgaris*.

Strain	*T. obliquus*		*C. vulgaris*	
Culture condition	1% v/v CO_2_	0.04% v/v CO_2_	1% v/v CO_2_	0.04% v/v CO_2_
Synthetic medium	154 ± 2^a^	137 ± 6^b^	58 ± 2^c^	62 ± 0^c^
Df 10	n.g.^1^	n.g.^1^	n.g.^1^	n.g.^1^
Df 15	34 ± 24^a^	n.g.^1^	12 ± 5^a^	n.g.^1^
Df 20	57 ± 8^a^	24 ± 18^b^	39 ± 11^ab^	17 ± 2^b^
Df 25	33 ± 18^a^	47 ± 8^a^	43 ± 12^a^	21 ± 3^a^

^1^ no growth.

Values in the same row that do not share the same letter indicate a statistically significant difference (*p*-value <0.05).

Furthermore, these results indicate that inorganic carbon did not limit the growth of either strain of microalgae under the tested conditions. The similar CO_2_ assimilation rates in synthetic medium cultures (both with and without CO_2_ injection) may also result from the microalgae not being acclimatized to CO_2_ injection prior to the experiments, as suggested by Azov (1982), since they were grown under laboratory conditions with 0.04% v/v CO_2_ beforehand.

In the synthetic medium, the CO_2_ assimilation rate values observed under both 0.04% and 1% v/v CO_2_ injection were comparable to those reported in the literature for *T. obliquus*. Ahiahonu et al. (2022) documented a CO_2_ assimilation rate of 0.265 ± 0.002 gCO_2_.L^-1^.d^-1^ for this microalgal strain, which exceeds that observed in our findings; their method involved initially injecting pure CO_2_ for 20 s before switching to air injection. Conversely, Chaudhary et al. (2018) reported a slightly lower CO_2_ assimilation rate for *T. obliquus* at 129.82 mg.L^-1^.d^-1^, using continuous injection of 5% v/v CO_2_. In the same study, they observed a higher CO_2_ assimilation rate for *C. vulgaris* (140.91 mg.L^-1^.d^-1^) than that obtained in the current research. Additionally, Kong et al. (2023) found that *C. vulgaris* could achieve CO_2_ assimilation rates ranging from 170.98 to 220.92 mgCO_2_.L^-1^.d^-1^, which also surpassed the results from this study. The authors noted that CO_2_ assimilation rates can vary based on the microalgal strain, the volume of CO_2_ injected, and the specific nutrients and components present in the culture medium.

Given the results obtained from the 200-mL cultures, the digestate diluted 15 times (Df 15) with an injection of 1% v/v CO_2_ was selected as the optimal culture condition for scaling up to 6 L.

### 3.2 Scale-up in flat panel reactors

#### 3.2.1 Growth kinetics and biomass production

**FIGURE 2:**
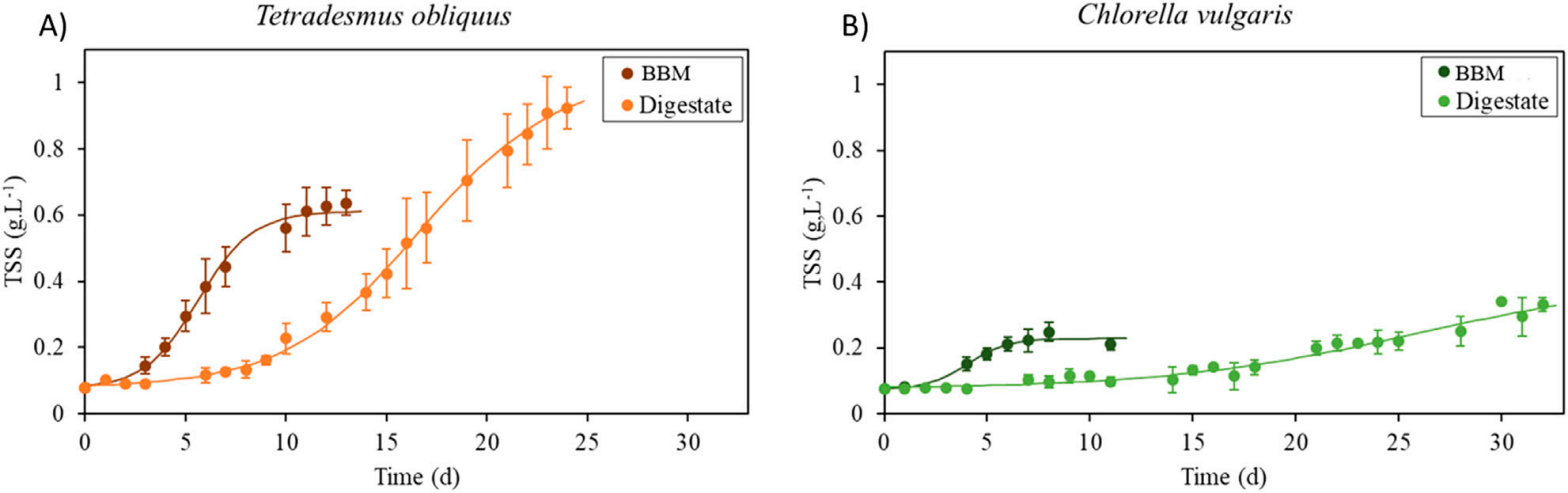
Growth curves of *T. obliquus* **(A)** and *C. vulgaris* **(B)** in 6-L flat panel bioreactors. Orange curves represent the biomass concentration (g.L^-1^) over the microalgal cultivation time of *T. obliquus,* and green curves represent the biomass concentration (g.L^-1^) of *C. vulgaris* over the microalgal cultivation time. Dark colors represent the BBM, and light colors correspond to the digestate culture medium.

*T. obliquus* and *C. vulgaris* microalgal strains were cultivated in 6-L bioreactors, and their growth kinetics, as well as biomass concentration and characterization properties, were assessed. A culture using the commercial BBM with continuous 1% v/v CO_2_ injection was carried out under the same scale conditions. The growth of the two microalgal strains was measured following the models of Equations 3 and 4 (Figure 2; Table 3), and the microalgal culture was monitored according to the following: (i) growth curve–microalgal biomass concentration; (ii) maximal growth rate (µ); (iii) final biomass concentration (iv) productivity; (v) the time of lag phase; (vi) the microalgal concentration (g.L^-1^) achieved by the culture just before entering in the stationary phase (X_0_.*e* ^(A)^); and (vii) the CO_2_ assimilation rate.

**TABLE 3:** Growth parameters of the 6-L cultures for both microalgal strains in the digestate diluted 15-times under 1% v/v CO_2_ injection. Values in the same row not followed by the same letter are significantly different (*p*-value <0.05).

Strain	*T. obliquus*		*C. vulgaris*	
Culture medium	Synthetic medium	Digestate	Synthetic medium	Digestate
µ max (d^-1^)	0.42 ± 0.15^ab^	0.13 ± 0.01^b^	0.87 ± 0.35^a^	0.06 ± 0.01^b^
Final algal biomass concentration (g.L^-1^)	0.61 ± 0.03^b^	0.92 ± 0.06^a^	0.23 ± 0.02^d^	0.33 ± 0.02^c^
Productivity (mg.L^-1^.d^-1^)	37.06 ± 0.00^a^	32.51 ± 0.00^a^	15.81 ± 0.00^b^	8.71 ± 0.00^c^
Lag-phase duration (d)	0.00 ± 0.00^b^	6.27 ± 0.89^a^	1.02 ± 0.32^b^	7.84 ± 0.92^a^
Plateaus (g.L^-1^)	0.61 ± 0.05^b^	1.03 ± 0.10^a^	0.23 ± 0.02^d^	0.42 ± 0.06^b^
CO_2_ assimilation rate (mgCO_2_.L^-1^.d^-1^)	59.75 ± 4.31^a^	58.37 ± 4.51^a^	28.97 ± 3.44^b^	14.67 ± 1.22^c^

Figure 2 shows the growth curves of *T. obliquus* and *C. vulgaris* in both the digestate and commercial medium. Notably, *T. obliquus* achieved a higher biomass concentration than *C. vulgaris* under both culture conditions. The acclimatization phase (lag phase) is longer in the digestate trials (lasting 6 days for *T. obliquus* and nearly 8 days for *C. vulgaris*) than in the synthetic medium assays (where the lag phase is effectively 0 days for *T. obliquus* and 1 day for *C. vulgaris*). This difference may stem from the strains being previously maintained in the laboratory using the same synthetic medium, requiring no adaptation. Conversely, the AD digestate presents challenges such as turbidity, elevated ammonium concentrations, and bacterial presence, necessitating a longer acclimatization phase for the microalgae to adapt. Despite this extended lag phase, *T. obliquus* demonstrates a shorter acclimatization time, leading to a more rapid transition into the exponential growth phase. Once acclimatized, it reaches a peak biomass concentration of 1.29 g.L^-1^, compared to just 0.36 g.L^-1^ for *C. vulgaris* under the same conditions. These findings align with those obtained by Fernandes et al. (2022), which also indicated that *T. obliquus* adapted more quickly to nitrogen sources (NH₄⁺) present in the digestate than *C. vulgaris*. Additionally, as shown in Figure 2, cultures in the digestate take longer to reach the stationary phase than those in the synthetic medium. This extended duration can be attributed to both the longer acclimatization phase and the higher nutrient content of the digestate-based media, which contains around three times more inorganic nitrogen than in the commercial BBM (See section 3.2.2).

The predicted times for both strains to reach their growth plateau, based on the growth model (Equation 4), exceeded the actual duration of the cultures. The cultivation of *T. obliquus* was halted just as it began approaching the plateau phase, while for *C. vulgaris*, it is difficult to pinpoint the exact growth stage as there is little differentiation between its growth phases on the graph.

Table 3 complements these results by providing key metrics: maximum growth rate, final algal biomass concentration, productivity, lag phase duration, plateau levels at the end of the culture, and the CO_2_ assimilation rates for each strain under synthetic medium and digestate conditions. Notably, *C. vulgaris* grown in the synthetic medium exhibits a significantly higher maximum growth rate (0.87 ± 0.35 d^-1^) than the other conditions: *T. obliquus* in the synthetic medium (0.42 ± 0.15 d⁻^1^), *T. obliquus* in the digestate (0.13 ± 0.01 d⁻^1^), and *C. vulgaris* in the digestate (0.06 ± 0.01 d⁻^1^). However, when considering the final microalgal biomass concentration, *T. obliquus* in the digestate performs significantly better (0.92 ± 0.06 g.L^-^1) than its growth in the synthetic medium (0.61 ± 0.03 g.L^-1^) and also surpasses *C. vulgaris* grown in both the synthetic medium (0.23 ± 0.02 g.L^-1^) and digestate (0.33 ± 0.02 g.L^-1^). In terms of productivity, *T. obliquus* shows no significant difference between its performance in the digestate (32.51 mg.L^-1^.d^-1^) and synthetic medium (37.06 mg.L^-1^.d^-1^). However, its productivity is significantly higher than that of *C. vulgaris,* which achieved 15.81 mg·L⁻^1^ d⁻^1^ in the synthetic medium and only 8.71 mg·L⁻^1^ d⁻^1^ in the digestate. As shown in Figure 2 and discussed previously, there is a significant difference in the lag phase between cultures grown in the synthetic medium and those grown in the digestate. The concentration at which the cultures reach the plateau phase, as predicted by the model (X_0_.*e* ^(A)^ in Equation 4), is represented in Table 3. *T. obliquus* grown in the digestate reached the highest concentration at this point (1.03 ± 0.10 g.L^-1^), significantly outperforming the other conditions. Regarding CO_2_ assimilation, a significant strain-specific difference was observed. *T. obliquus* in the synthetic medium (59.75 ± 4.31 mgCO_2_.L^-1^.d^-1^) and digestate (58.37 ± 4.51 mgCO_2_.L^-1^.d^-1^) assimilated more CO_2_ than *C. vulgaris* in the synthetic medium (28.97 ± 3.44 mgCO_2_.L^-1^.d^-1^). *C. vulgaris* assimilated even less CO_2_, when grown in the digestate (14.67 ± 1.22 mgCO_2_.L^-1^.d^-1^) compared to synthetic medium. According to Fernandes et al. (2022), lowering the pH of the medium could have a negative, stressful effect on *C. vulgaris* growth but a positive impact on *T. obliquus*, which aligns with the current study findings, where CO₂ injection lowered the pH. Additionally, the reduced growth of *C. vulgaris* may be related to its reduced ability to fix NH_4_^+^ compared to *T. obliquus* under these conditions. A lower rate of nitrogen assimilation translates into reduced protein synthesis, which is vital for DNA replication and protection. The N/P molar ratio in this medium is 57, which deviates greatly from the ideal ratio of 8, as suggested by Xie et al. (2023). This imbalance may further hinder nitrogen uptake by *C. vulgaris*.

Overall, all growth parameters indicate that *T. obliquus* exhibited superior growth performance compared to *C. vulgaris* in the digestate diluted 15-fold with 1% v/v CO₂ injection, in terms of both productivity (biomass concentration) and adaptability to the cultivation environment.

#### 3.2.2 Bioremediation potential

In order to calculate the capacity of the two microalgae to use some residual nutrients and organic matter present in the digestate, the concentrations of COD, P-PO_4_^3-^, and inorganic nitrogen species were measured in the digestate-based media at the beginning and end of each culture (Table 4).

**TABLE 4:** Initial (Ci) and final (Cf) concentrations of different parameters measured in the AD liquid digestate at the beginning and the end of the culture for both microalgae strains.

Parameters	*T. obliquus*		*C. vulgaris*	
	C_i_ (mg.L^-1^)	C_f_ (mg.L^-1^)	C_i_ (mg.L^-1^)	C_f_ (mg.L^-1^)
COD	1392 ± 60	706 ± 25	1265 ± 5	612 ± 13
P-PO_4_^3-^	26.1 ± 1.1	12.4 ± 0.5	6.5 ± 0.2	2.8 ± 0.5
TIN	129.2 ± 6.3	6.2 ± 1.0	149.2 ± 0.8	6.5 ± 2.5
N-NH_4_^+^	127.9 ± 6.0	0.2 ± 0.2	148.0 ± 0.8	0.4 ± 0.4
N-NO_3_^-^	0.7 ± 0.3	1.7 ± 0.3	0.5 ± 0.0	2.2 ± 1.0
N-NO_2_^-^	0.6 ± 0.0	4.3 ± 0.8	0.7 ± 0.0	4.0 ± 1.4

**FIGURE 3:**
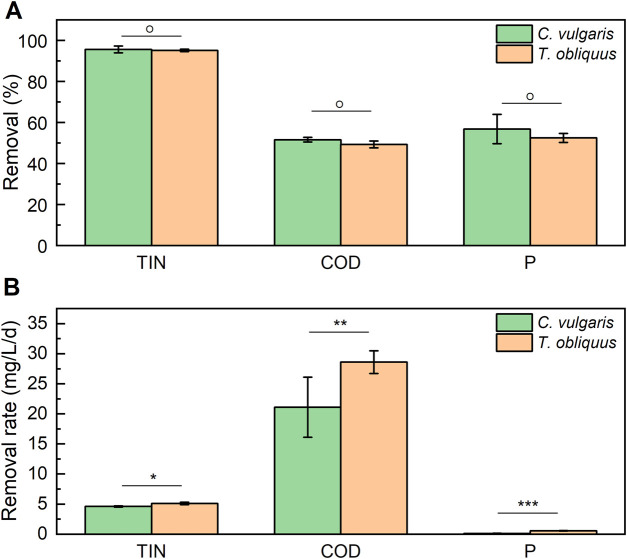
Total removal **(A)** and removal rate **(B)** of total inorganic nitrogen (TIN), chemical oxygen demand (COD), and phosphates (P) by *T. obliquus* (orange) and *C. vulgaris (green)* microalgae. Both strains were cultivated in 6-L bioreactors using diluted digestate as culture media (Df 15) supplemented with 1% v/v CO_2_. Significant differences are indicated based on one-way analysis of variance results: *: *p* < 0.05; **: *p* < 0.01; ***: *p* < 0.001; and °: *p* > 0.05.

Both strains demonstrated nearly complete TIN removal, achieving almost 100% elimination. Additionally, they removed approximately 55% of phosphate (P-PO₄³⁻) and 50% of COD, with no significant difference observed between the two strains (Figure 3). The highest removal rates were observed for COD, with *T. obliquus* and *C. vulgaris* achieving rates of 28.60 ± 1.90 and 21.10 ± 0.50 mg.L^-1^.d^-1^, respectively. In contrast, phosphate removal rates were the lowest, with *T. obliquus* removing 0.57 ± 0.04 mg·L⁻^1^ d⁻^1^ and *C. vulgaris* removing 0.12 ± 0.02 mg·L⁻^1^ d⁻^1^. Regarding TIN removal, *T. obliquus* achieved a rate of 5.10 ± 0.20 mg·L⁻^1^ d⁻^1^, while *C. vulgaris* showed a slightly lower rate of 4.60 ± 0.1 mg·L⁻^1^ d⁻^1^. No significant difference was found between the two microalgal strains in their ability to remove any of the pollutants measured (% removal), indicating that both strains exhibit similar bioremediation results under these conditions. Nevertheless, when measuring the removal rate capacity of the two microalgae for the three nutrients, significant differences were found (Figure 3), observing *C. vulgaris* having higher removal rate values for the three measured nutrients.

The experimental results suggest that microalgae were capable of efficiently assimilating N-NH_4_^+^ from the digestate as a source of nitrogen, in line with previous studies (Uggetti et al., 2014). However, the slight increase in N-NO_3_^-^ and N-NO_2_^-^ concentrations observed by the end of the cultivation indicates the possible action of nitrifying bacteria (Table 4) (Sánchez-Zurano et al., 2021). The final concentration of inorganic nitrogen species is still very low, with 6.5 ± 2.5 and 6.2 ± 1.0 mgN.L^-1^ detected in the digestate-based media used for the cultivation of *C. vulgaris* and *T. obliquus*, respectively. Such decontamination enhances the potential for the treated effluent to be reused, as one of the key environmental concerns with using untreated digestate as a soil amendment is its excessive N content (Doyeni et al., 2021).

As reported by Marcilhac et al. (2015), phosphorus concentration is less critical for microalgal growth compared to nitrogen. This is consistent with the findings in this study, where the microalgae ceased growing once N-NH_4_^+^ was depleted, even though the P-PO_4_^3-^ levels remained. Additionally, the data suggest that the microalgae possibly employed a mixotrophic metabolism, as proposed by Bentahar and Deschênes (2022) and Manhaeghe et al. (2020). This type of metabolism allows the microalgae to consume part of the organic carbon from the digestate without hindering the fixation of inorganic carbon supplied by CO_2_ injection (1%), thereby enabling the simultaneous decontamination of both liquid and gaseous effluents.

According to the nitrogen mass balance analysis, it was observed that only part of the TIN removed was attributed to the fixation of nitrogen in the microalgal biomass. In the case of *C. vulgaris*, 14.9% of the inorganic nitrogen eliminated during the cultivation was detected in the final biomass, which was similar to the other study where the nitrogen mass balance was 11.8% (Barreiro-Vescovo et al., 2020) The assimilation of nitrogen was higher in the case of *T. obliquus* (45.0%), which was mainly explained by the higher biomass productivity and TIN removal rate obtained in these conditions. This value is close to those reported by Romero-Villegas et al. (2018) with values between 21% and 73%. The authors cultivated *Nannochloropsis gaditana* in an outdoor semicontinuous culture using as the culture medium a centrate from a wastewater treatment plant diluted with seawater.

Consequently, it can be assumed that a large fraction of the inorganic nitrogen was lost by another process than biofixation by microalgae cells, the most likely being ammonia stripping, accounting for 55.0% and 85.1% for *T. obliquus* and *C. vulgaris* cultures, respectively (Barreiro-Vescovo et al., 2020). Even if the continuous injection of 1% v/v CO_2_ in the culture media maintained the pH at approximately 8.0, volatilization of ammonia is a common phenomenon observed during the long period of cultivation with the anaerobic digestate (Fernandes et al., 2022).

#### 3.2.3 Assessment of the biochemical composition of the harvested biomass

Once the microalgal biomass was harvested and freeze-dried, it was characterized for various biochemical components, including (i) lipids, (ii) monomeric sugars, (iii) chlorophylls, (iv) soluble and total proteins, (v) total phenolic compounds, (vi) auxins, (vii) antioxidant potential, (viii) minerals/metals, and (ix) ash content. Previous studies have shown that the biochemical composition of microalgal strains can vary depending on genetic factors, culture conditions, and the growth phase during analysis (Chen and Wang, 2021). The objective here was to compare the biomass composition of *T. obliquus* and *C. vulgaris* under two specific culture conditions: (i) digestate, where the microalgae exhibit a mixotrophic metabolism, and (ii) synthetic medium, where the microalgae follow an autotrophic metabolism.

Both strains cultivated in the digestate exhibited a low lipid content, with *T. obliquus* containing 9.97% ± 2.48% DW and *C. vulgaris* containing 9.53% ± 0.87% DW (Table 5). These values are lower than those previously reported by Koutra et al. (2021), where *C. vulgaris* grown in 10% diluted AD liquid digestate reached approximately 16% DW. The lipid content of *C. vulgaris* in the synthetic medium reached up to 21.24% ± 0.96% DW, more than double that of the digestate. This discrepancy could be attributed to the higher ammonium levels in the digestate compared to the synthetic medium as elevated ammonium levels typically lead to reduced carbohydrate and lipid accumulation while promoting protein synthesis (Li et al., 2019).

**TABLE 5:** Biomass composition of *T. obliquus* and *C. vulgaris* cultivated in the synthetic medium and AD digestate.

Parameter	Units	*T. obliquus*		*C. vulgaris*	
		Synthetic medium	Digestate	Synthetic medium	Digestate
Ash	% DW	11.50 ± 2.00^a^	5.20 ± 1.00^bc^	3.90 ± 2.00^c^	8.60 ± 1.00^ab^
Lipids	% DW	10.19 ± 0.75^b^	9.97 ± 2.48^b^	21.24 ± 0.96^a^	9.53 ± 0.87^b^
Carbohydrates	% DW	41.10 ± 4.60^a^	9.00 ± 1.90^bc^	14.50 ± 2.60^b^	5.10 ± 0.30^c^
Chlorophylls	% DW	1.00 ± 0.39^a^	0.41 ± 0.13^ab^	0.81 ± 0.13^ab^	0.32 ± 0.14^b^
Total proteins	% DW	16.12 ± 1.50^b^	46.21 ± 3.98^a^	17.45 ± 1.26^b^	44.17 ± 2.24^a^
Extractable proteins	% DW	9.40 ± 0.04^b^	29.30 ± 0.04^a^	14.40 ± 0.03^b^	23.90 ± 0.05^a^
Nitrogen (N)	% DW	2.79 ± 0.20^b^	7.07 ± 0.36^a^	2.59 ± 0.24^b^	7.60 ± 0.64^a^
Carbon (C)	% DW	44.18 ± 1.74^a^	49.14 ± 2.47^a^	51.93 ± 3.34^a^	46.35 ± 2.34^a^
Hydrogen (H)	% DW	7.44 ± 0.24^ab^	7.42 ± 0.34^ab^	8.24 ± 0.38^a^	7.07 ± 0.34^b^
Antioxidants (FRAP)	µg Trolox. mg DW^-1^	1.85 ± 0.23^ab^	2.41 ± 0.14^ab^	2.78 ± 0.53^a^	1.98 ± 0.21^b^
Total phenolic compounds	% DW	0.16 ± 0.11^a^	0.15 ± 0.08^a^	0.15 ± 0.07^a^	0.05 ± 0.02^a^
Auxins	µg IAA.g DW^-1^	0.00 ± 0.00^b^	0.00 ± 0.00^b^	0.00 ± 0.00^b^	5.59 ± 1.57^a^

Values within the same row that do not share the same letter indicate a statistically significant difference (*p*-value <0.05).

Similarly, the carbohydrate content was significantly higher in the biomass cultivated in the synthetic medium compared to the digestate for both strains. *T. obliquus* in the synthetic medium reached 41.10% ± 4.60% DW—double the amount found in *C. vulgaris* (14.50% ± 2.60% DW)—while in the digestate, *T. obliquus* recorded only 9.00% ± 1.90% DW (with *C. vulgaris* reaching 5.10% ± 0.30% DW). The marked reduction in the carbohydrate content in digestate-grown strains is likely associated to the higher organic carbon content in the digestate (COD = 1400 mg.L^-1^) than in the synthetic medium. According to Piasecka et al. (2020), autotrophic microalgae tend to produce and accumulate more carbohydrates than those grown mixotrophically, which aligns with the present study’s results comparing autotrophic growth in the synthetic medium to mixotrophic growth in the digestate.

Both total and hydrosoluble protein contents were strongly influenced by the culture medium. The total protein content of both strains was more than 2.5 times higher in biomass cultivated in digestate compared to the synthetic medium, with *T. obliquus* reaching 46.2% DW in digestate and 16.1% DW in the synthetic medium, while *C. vulgaris* had 44.2% DW in digestate and 17.5% DW in the synthetic medium. These findings confirm that digestate favors protein production at the expense of carbohydrates and lipids. Regarding the synthetic culture medium, it is possible that the low initial concentration of nitrates quickly led to nitrogen starvation, affecting significantly the metabolism of microalgae.

The high turbidity of the diluted digestate combined with its rich organic carbon content likely contributed to reduced photosynthetic capacity in the microalgae strains (Li et al., 2020). This reduction is reflected in the significantly lower chlorophyll content observed in both strains grown in digestate, with *T. obliquus* and *C. vulgaris* recording 4.11 ± 1.34 µg total chlorophylls.mg DW⁻^1^ and 3.22 ± 1.38 µg total chlorophylls.mg DW⁻^1^, respectively. In contrast, when grown in the synthetic medium, chlorophyll levels were notably higher at 9.50 ± 3.88 µg total chlorophylls.mg DW⁻^1^ for *T. obliquus* and 8.075 ± 1.32 µg total chlorophylls.mg DW⁻^1^ for *C. vulgaris*. This trend is consistent with the findings of Li et al. (2020), who observed a similar reduction in chlorophyll content for *Asterarcys* sp. when cultivated mixotrophically rather than autotrophically. In their study, a lower chlorophyll concentration was suggested to indicate that the cells were less reliant on solar energy in mixotrophic conditions.

The monomeric sugar profiles obtained after acid hydrolysis differed between the two strains (Supplementary Table S3). In *T. obliquus,* only glucose was detected, while *C. vulgaris* exhibited both glucose and rhamnose, with rhamnose being present in smaller quantities than glucose, consistent with observations by Ortiz-Tena et al. (2016). No glucuronic or galacturonic acids were identified during the HPLC assay.

To further explore the potential of the microalgal biomass as plant biostimulants, several bioactive components were evaluated, including antioxidant activity, phenolic content, and the presence of phytohormones like auxins (IAA) or other molecules with phytohormone-like activity.

Table 5 reveals that the highest antioxidant potential (measured using the FRAP assay) was observed in *C. vulgaris* cultivated in synthetic medium. While the FRAP test indicated positive antioxidant activity for all samples, the levels were relatively modest. Specifically, *T. obliquus* demonstrated an antioxidant activity of 1.85 ± 0.23 µg Trolox/mg DW in synthetic medium and 2.78 ± 0.53 µg Trolox/mg DW in digestate. For *C. vulgaris*, the values were 2.41 ± 0.14 µg Trolox/mg DW in the synthetic medium and 1.98 ± 0.21 µg Trolox/mg DW in the digestate.

According to Hajimahmoodi et al. (2010), research on the correlation between total phenolic content and antioxidant capacity is still limited; however, their findings suggest a direct relationship between antioxidants identified through the FRAP method and total phenolic content. In our study, both strains contained phenolic compounds, albeit in low concentrations: *T. obliquus* exhibited values of 0.16% ± 0.11% DW in the synthetic medium and 0.15% ± 0.08% DW in the digestate, while *C. vulgaris* showed values of 0.15% ± 0.07% DW in the synthetic medium and 0.05% ± 0.02% DW in the digestate.

In terms of auxin-like molecule production, only *C. vulgaris* grown in the digestate synthesized IAA-like molecules, as shown in Table 5. These phytohormones play a crucial role in promoting microalgal growth and enhancing production yields. However, the biosynthetic pathways for IAA or IAA-like molecules in microalgae remain undetermined (Lin et al., 2022).

*T. obliquus* cultured in the synthetic medium exhibited a significantly higher ash content of 11.50% ± 2.00% DW compared to the other samples. Specifically, *T. obliquus* in the digestate showed 5.20% ± 1.00% DW, while *C. vulgaris* had ash contents of 3.90% ± 2.00% DW in the synthetic medium and 8.60% ± 1.00% DW in the digestate. This trend corresponds to the two to four times higher phosphorus content and 1.5 times higher calcium content in this strain and medium relative to *T. obliquus* in the digestate and *C. vulgaris* in both media (Table 6). The second highest ash content was observed in *C. vulgaris* grown in the digestate, with a value of 8.60% ± 1.00% DW. This can be attributed to the substantial amounts of metals present, including Fe (907.64 ± 40.04 mg/kg DW), Mn (204.30 ± 27.68 mg/kg DW), and Ni (4.26 ± 0.31 mg/kg DW) (Table 6). As noted in Supplementary Table S2, the liquid digestate had elevated concentrations of certain metals, such as Cu (2400 μg/L), Mn (1600 μg/L), and Zn (9600 μg/L), compared to the BBM. All observed ash content values fell within the ranges reported in the literature (Fox and Zimba, 2018). The microalgae cultivated in this effluent may have developed specific defense mechanisms to limit metal uptake (Chen D. et al., 2023), such as the synthesis and secretion of exoproteins (Expósito et al., 2021). This could also help explain the significant differences in the protein content observed in these microalgae when cultivated in the digestate (Table 5). Table 6 shows that *C. vulgaris* accumulates more metals than *T. obliquus* when grown in the digestate, which may account for the lower growth rates of *C. vulgaris* compared to *T. obliquus* in this alternative culture medium (Figure 2). It is possible that *C. vulgaris* has bioaccumulated or biosorbed more metals than *T. obliquus* (Yeheyo et al., 2024), highlighting the species-specific sensitivity to various contaminants, including metals (Expósito et al., 2021).

**TABLE 6:** Metal content measured by ICP-MS in *T. obliquus* and *C. vulgaris* cultivated in the synthetic medium and AD digestate.

Compound	Units	*T. obliquus*		*C. vulgaris*		Biostimulant legislation^2^
		Synthetic medium	Digestate	Synthetic medium	Digestate	
Mg	g.kg DW^-1^	1.58 ± 0.18^b^	2.32 ± 0.16^a^	1.80 ± 0.15^b^	2.23 ± 0.31^a^	-
P	g.kg DW^-1^	21.67 ± 4.64^a^	9.16 ± 1.17^b^	12.42 ± 0.63^b^	8.90 ± 0.73^b^	-
K	g.kg DW^-1^	9.20 ± 0.21^a^	8.05 ± 0.51^b^	8.71 ± 0.36^ab^	8.82 ± 0.37^ab^	-
Ca	g.kg DW^-1^	23.82 ± 9.06^a^	6.14 ± 0.68^b^	15.39 ± 0.67^ab^	13.42 ± 1.35^ab^	-
Cr	mg.kg DW^-1^	0.49 ± 0.09^c^	2.22 ± 0.18^b^	1.38 ± 0.88^bc^	3.54 ± 0.30^a^	2.00
Fe	mg.kg DW^-1^	238.51 ± 46.24^c^	616.56 ± 72.15^b^	101.87 ± 15.45^d^	907.64 ± 40.04^a^	-
Mn	mg.kg DW^-1^	120.52 ± 44.30^b^	120.82 ± 13.21^b^	37.56 ± 2.88^c^	204.30 ± 27.68^a^	-
Ni	mg.kg DW^-1^	2.06 ± 0.69^c^	3.25 ± 0.13^b^	1.04 ± 0.05^d^	4.26 ± 0.31^a^	50.00
Cu	mg.kg DW^-1^	57.94 ± 9.69^b^	93.84 ± 12.16^a^	12.63 ± 1.59^c^	107.91 ± 6.47^a^	600.00
Zn	mg.kg DW^-1^	89.85 ± 23.21^c^	217.42 ± 18.13^a^	25.25 ± 1.63^d^	174.36 ± 9.23^b^	1500.00
As	mg.kg DW^-1^	0.06 ± 0.01^c^	1.01 ± 0.03^a^	0.02 ± 0.00^c^	0.32 ± 0.01^b^	40.00
Cd	mg.kg DW^-1^	<LOD^1^	1.01×10^−2^ ± 2.17×10^−3a^	<LOD^1^	8.98×10^−3^ ± 1.41×10^−3a^	1.50
Hg	mg.kg DW^-1^	<LOD^1^	<LOD^1^	<LOD^1^	<LOD^1^	1.00
Pb	mg.kg DW^-1^	0.12 ± 0.03^c^	0.39 ± 0.11^b^	0.12 ± 0.06^c^	0.71 ± 0.04^a^	120.00

^1^ Limit of detection—Cd: 0.003 mg.kg DW_-1_; Hg: 0.0223 mg.kg DW_-1_.

^2^ (Regulation (EU) 2019/1009 of the European Parliament and of the Council of 5 June 2019 Laying down Rules on the Making Available on the Market of EU Fertilizing Products and Amending Regulations (EC) No 1069/2009 and (EC) No 1107/2009 and Repealing Regulation (EC) No 2003/2003 (Text with EEA Relevance), 2019).

Values within the same row that do not share the same letter indicate a statistically significant difference (*p*-value <0.05).

Álvarez-González et al. (2023a) also investigated the biostimulant potential of microalgae cultivated in liquid effluents, yielding positive results. Their findings regarding biomass composition are comparable to our own, as they report protein levels of 38%–48% DW (with our study showing 44%–46% DW) and carbohydrate concentrations of 9%–19% DW (5%–9% DW in our research).

Regarding the metals detected in the microalgae biomass, only Cr slightly exceeded the permissible limit, recorded at 0.22 mg kg DW^-1^ for *T. obliquus* and 1.54 mg.kg DW^-1^ for *C. vulgaris*. The concentrations of other metals fell within regulatory limits established for using this biomass as a biostimulant and biofertilizer in the EU (Regulation (EU) 2019/1009 of the European Parliament and of the Council of 5 June 2019 Laying down Rules on the Making Available on the Market of EU Fertilizing Products and Amending Regulations (EC) No 1069/2009 and (EC) No 1107/2009 and Repealing Regulation (EC) No 2003/2003 (Text with EEA Relevance), 2019) (Table 6). In a prior study by Álvarez-González et al. (2023b), which focused on cultivating the same microalgae in municipal wastewater for potential agricultural biostimulants, the only metal to exceed regulatory thresholds was Cd, with no violations for Cr. It is worth mentioning that the downstream processing of the algal biomass usually includes the use of disruptive methods and solid/liquid extraction techniques, which might reduce the presence of metals in the final extracts. Therefore, it is essential to measure Cr concentrations in future biostimulant extracts derived from the biomass cultivated in the digestate to ensure compliance with existing legislation.

These results demonstrate that both the selection of the microalgal strain and the culture conditions influence their biochemical constitution. The source and concentration of nitrogen in the medium have significantly impacted the protein content, which in turn influences the lipid and carbohydrate levels in the microalgae studied. Both microalgae—*T. obliquus* and *C. vulgaris*—cultivated in the digestate and synthetic medium, show promise as candidates for the extraction of plant biostimulants. Specifically, those grown in the synthetic medium are particularly valuable for their carbohydrate content, while those cultivated in the digestate are noteworthy for their higher protein composition, along with beneficial mineral content and antioxidant potential. Moreover, the presence of IAA in *C. vulgaris* grown in the digestate could further enhance plant growth (Sánchez-Quintero et al., 2023).

## 4 Conclusion and perspectives

The biomass produced holds potential as biostimulants and/or biofertilizers for sustainable agriculture, supported by its high protein content derived from AD digestate cultivation. Future extraction processes may yield products with reduced chromium levels compared to the whole biomass, warranting further analysis *via* ICP-MS to ensure compliance with legislative thresholds.

To assess the practical application of these extracts as biostimulants, additional plant growth tests are necessary. Furthermore, any residual biomass with lower biostimulant potential can be redirected toward other applications, such as lipid extraction or the isolation of structural cell wall components.

In summary, both *T. obliquus* and *C. vulgaris* demonstrated effective nutrient uptake from AD liquid digestate diluted 15 times with 1% v/v CO_2_ injection. *T. obliquus* proved to be more efficient in terms of productivity and adaptation to the growth environment, highlighting the significant influence of culture media composition on the biochemical profile of these microalgae.

## Data Availability

The original contributions presented in the study are included in the article/Supplementary Material; further inquiries can be directed to the corresponding author.
