# Supplementary material for: Effects of CO2 and liquid digestate concentrations on the growth performance and biomass composition of Tetradesmus obliquus and Chlorella vulgaris microalgal strains
Source: Front Bioeng Biotechnol. 2025 Jan 9;12:1459756. doi: 10.3389/fbioe.2024.1459756 (PMC11755043; doi:10.3389/fbioe.2024.1459756)
Supplement: Supplementary file 1 [file DataSheet1.docx]

**Table S1**: Physico-chemical composition of the raw anaerobic digestate straight after collection.

| **Parameters** | **Unit** | **Value** |
| --- | --- | --- |
| **General** |  |  |
| Dry matter | % | 7 |
| Humidity | % | 93 |
| Conductivity | dS·m^-1^ | 5.1 |
| Mineral matter | g·100 g MS^-1^ | 28.7 |
| Organic matter | g·100 g MS^-1^ | 71.3 |
| Organic carbon | g·100 g MS^-1^ | 35.6 |
| pH | - | 8.7 |
| Turbidity | FNU | 12840 |
| **Agronomic properties** |  |  |
| Kjeldahl nitrogen | g·100 g MS^-1^ | 6.74 |
| Organic Nitrogen | g·100 g MS^-1^ | 4.74 |
| Ammoniacal Nitrogen | g·100 g MS^-1^ | 2.01 |
| C/N ratio | - | 5.3 |
| Phosphorus | g·100 g MS^-1^ | 3.61 |
| Potassium | g·100 g MS^-1^ | 7.33 |
| Calcium | g·100 g MS^-1^ | 3.97 |
| Magnesium | g·100 g MS^-1^ | 1.66 |
| Sulphur | g·100 g MS^-1^ | 1.23 |
| Sodium | g·100 g MS^-1^ | 0.95 |

**Table S2**. Physico-chemical composition of the anaerobic liquid digestate after phase separation.

| **Parameters** | **Unit** | **Value** |
| --- | --- | --- |
| **General** |  |  |
| pH |  | 8.23 |
| Conductivity | mS.cm^-1^ | 24.8 |
| **Elements** |  |  |
| COD^1^ | mg.L^-1^ | 19 700 |
| Total VFA^2^ | mg.L^-1^ | 179 |
| Total Nitrogen (N) | mg.L^-1^ | 3 700 |
| N-NH_4_^+^ | mg.L^-1^ | 1 875 |
| N-NO_3_^-^ | mg.L^-1^ | 123 |
| Total Phosphorus (P) | mg.L^-1^ | 140 |
| P-PO_4_^3-^ | mg.L^-1^ | 99 |
| Sodium (Na) | mg.L^-1^ | 500 |
| Magnesium (Mg) | mg.L^-1^ | 30 |
| Potassium (K) | mg.L^-1^ | 4 100 |
| Calcium (Ca) | mg.L^-1^ | 97 |
| Chrome (Cr) | µg.L^-1^ | <130 |
| Manganese (Mn) | µg.L^-1^ | 1 600 |
| Iron (Fe) | mg.L^-1^ | 47 |
| Cobalt (Co) | µg.L^-1^ | <250 |
| Nickel (Ni) | µg.L^-1^ | 310 |
| Copper (Cu) | µg.L^-1^ | 2 400 |
| Zinc (Zn) | µg.L^-1^ | 9 600 |
| Arsenic (As) | µg.L^-1^ | <75 |
| Molybdenum (Mo) | µg.L^-1^ | <250 |
| Cadmium (Cd) | µg.L^-1^ | <38 |
| Mercury (Hg) | µg.L^-1^ | <13 |
| Lead (Pb) | µg.L^-1^ | <250 |
| Silver (Ag) | µg.L^-1^ | <10 |

^1^Chemical Oxygen Demand; ^2^Volatile Fatty Acids

**Table S3**: Monomeric sugars detected in *T. obliquus* and *C. vulgaris* hydrolysates following the NREL protocol presented in section 2.4.4. Results are expressed as % of DW for both strains cultivated in synthetic media and diluted digestate.

| Conditions | | Glucose | Rhamnose |
| --- | --- | --- | --- |
| *T. obliquus* | Synthetic media | 41.1 ± 4.6 | 0.0 ± 0.0 |
|  | Digestate | 9.0 ± 1.9 | 0.0 ± 0.0 |
| *C. vulgaris* | Synthetic media | 12.7 ± 2.5 | 1.4 ± 0.5 |
|  | Digestate | 3.9 ± 0.5 | 1.0 ± 0.1 |
